# Carnosine Potentiates a Compensatory Mitochondrial–Synaptic Proteomic Response in the ALS Cerebellum

**DOI:** 10.3390/antiox15091117

**Published:** 2026-09-04

**Authors:** Hellen P. Valerio, Valeria Oliveira, Stephanie Y. Ferreira, Isabel R. Pereira, Giuseppe Palmisano, Mariana P. Massafera, Vanderson S. Bispo, Fernanda M. Prado, Paolo Di Mascio, Marisa H. G. Medeiros

**Affiliations:** 1 GlycoProteomics Laboratory, Department of Parasitology, ICB, Universidade de São Paulo, São Paulo 05508-000, Brazil; hellen.valerio@usp.br (H.P.V.); palmisano.gp@usp.br (G.P.); 2 Departamento de Bioquímica, Instituto de Química, Universidade de São Paulo, São Paulo 05508-000, Brazil; valeria.oliveira@usp.br (V.O.); stephanieyaciche@usp.br (S.Y.F.); isabel09@usp.br (I.R.P.); m.massafera-pereira@lesaffre.com (M.P.M.); vanderson.bispo@ephar.com.br (V.S.B.); fmprado@iq.usp.br (F.M.P.); pdmascio@iq.usp.br (P.D.M.)

**Keywords:** amyotrophic lateral sclerosis, carnosine, cerebellum, proteomics, SOD1^G93A^

## Abstract

Amyotrophic lateral sclerosis (ALS) is a fatal neurodegenerative disease characterized by progressive motor neuron degeneration and chronic neuroinflammation in the brain and spinal cord, involving complex interactions between neurons and immune cells. Carnosine (β-alanyl-L-histidine) has pathophysiological relevance due to its ability to detoxify reactive carbonyl species, including α,β-unsaturated aldehydes, scavenge free radicals, and chelate zinc, and has also been proposed to function in the central nervous system as a histidine reservoir for histamine synthesis. Here, we investigated the effects of carnosine supplementation on the cerebellar proteome of SOD1^G93A^ ALS rats using quantitative proteomics. Carnosine treatment extensively remodeled mitochondrial, antioxidant, and synaptic vesicle-trafficking protein networks and increased the abundance of glutamatergic and GABAergic receptor subunits relative to untreated ALS animals, with several of these changes exceeding wild-type levels. Pathway enrichment analyses identified significant up-regulation of Rab-mediated vesicle trafficking, synaptic vesicle cycling, and neurotransmitter transport/secretion pathways, alongside a partial reduction in RNA splicing and proteasomal subunits that were elevated in untreated ALS animals. Cross-comparison with the ALS-associated proteomic signature revealed that most carnosine-responsive proteins followed, rather than reversed, the direction of disease-associated change, indicating that carnosine predominantly potentiates an endogenous compensatory program rather than restoring a wild-type-like proteome. Collectively, these findings show that carnosine drives systems-level remodeling of mitochondrial and synaptic networks in the ALS cerebellum, identifying candidate compensatory pathways and supporting further functional validation of carnosine as a component of multimodal therapeutic strategies in ALS.

## 1. Introduction

Amyotrophic lateral sclerosis (ALS) is a fatal neurodegenerative disease characterized by progressive motor neuron loss, leading to muscle atrophy, paralysis, and death [1]. Although most cases are sporadic and of unknown origin, approximately 10% are familial [2], and a substantial proportion of these are associated with mutations in the superoxide dismutase 1 (SOD1) gene [3]. Transgenic SOD1^G93A^ models [4] reproduce key pathological features of ALS, including protein aggregation, oxidative stress, mitochondrial dysfunction, and synaptic abnormalities [5,6]. Mitochondrial dysfunction represents a central pathogenic axis in ALS [7]. Impairments in oxidative phosphorylation, calcium buffering, mitochondrial dynamics, and protein import contribute to bioenergetic failure and neuronal vulnerability [8,9,10]. In parallel, synaptic dysfunction is increasingly recognized as an early and critical event in disease progression [11]. Disruption of vesicle trafficking, Rab GTPase signaling, and autophagy–lysosomal pathways compromises neurotransmitter release and intracellular proteostasis [12,13]. Currently, ALS is recognized as a multisystem disorder characterized by structural and metabolic abnormalities across multiple cell types that synergistically accelerate disease progression [14]. Although the cerebellum was previously not considered to be involved in ALS, growing neuropathological evidence now supports its participation [15]. Neuroimaging studies using advanced MRI techniques have demonstrated that multiple cerebral regions, including the cerebellum, are recruited to compensate for motor functional decline [16]. More recently, resting-state functional connectivity changes in both motor and extra-motor cerebellar regions were identified in patients with sporadic ALS (sALS) [17]. Although the toxic mechanisms of mutant SOD1 in ALS remain incompletely understood, the active propagation of pathology through lipid peroxidation during neuroinflammation is likely a central component of disease progression. Endogenously, reactive aldehydes, including *trans*-4-hydroxynonenal (HNE), are formed as secondary lipid peroxidation products [18]. Oxidative stress biomarkers like 3-nitrotyrosine, 8-hydroxy-2′-deoxyguanosine and HNE have been found to be elevated in ALS patients [19]. Additionally, HNE-protein adducts have been observed to be elevated in brain tissue and body fluids in various age-related neurodegenerative diseases, including Alzheimer’s disease, Parkinson’s disease, Huntington’s disease, and ALS, as well as in corresponding disease models [20]. Consequently, antioxidant therapies have been proposed for ALS treatment. However, the limited efficacy of those therapies is largely attributed to pathological alterations in the blood–brain barrier, which restrict central nervous system drug penetration [21]. Histidine-containing dipeptides, such as carnosine (β-alanyl-L-histidine, Car), are present at high concentrations in skeletal muscle and the central nervous system and can readily cross the blood–brain barrier [22], making them attractive candidates for CNS-targeted antioxidant therapy. We hypothesized that carnosine supplementation could mitigate ALS-associated dysfunctions. To test this hypothesis, we performed an integrated quantitative proteomic analysis of cerebellar tissue from SOD1^G93A^ rats with or without carnosine supplementation.

## 2. Materials and Methods

Animals and experimental design. All animal procedures were conducted in accordance with the ethical principles for animal experimentation and complied with the guidelines of the National Council for the Control of Animal Experimentation (CONCEA, Ministry of Science, Technology and Innovation, Brasília, Brazil). The study protocol was approved by the Animal Care and Use Committee of the Institute of Chemistry, University of São Paulo (protocol no. 194/2021). Male hemizygous Sprague–Dawley rats overexpressing human SOD1^G93A^, carrying a glycine-to-alanine substitution at residue 93 of the SOD1 protein (strain NTac:SD-Tg(SOD1^G93A^)L26H), a well-established rodent model of ALS, were obtained from Taconic and bred with wild-type Sprague–Dawley females to establish the colony. These animals express human SOD1^G93A^ in the spinal cord at levels approximately eight-fold higher than endogenous SOD1, although the transgene is expressed ubiquitously across tissues. Genotyping was performed by PCR to detect the human SOD1 transgene using genomic DNA extracted from ear-punch tissue samples collected at 20 days of age. Animals were housed under controlled laboratory conditions (12 h light/12 h dark cycle, constant room temperature) with food and water available *ad libitum*.

### 2.1. Experimental Design

Male rats (n = 36) were assigned to four groups: WT (untreated), WT treated with carnosine (WT_CAR), ALS (untreated), and ALS treated with carnosine (ALS_CAR). Starting at 60 days of age, the treated groups, namely WT_CAR (n = 9) and ALS_CAR (n = 9), received L-carnosine (1 mg/mL; Sigma, Cat. No. C9625) in the drinking water, whereas the untreated groups, WT (n = 8) and ALS (n = 9), received regular drinking water. Water intake was monitored three times per week, and no statistically significant differences in total consumption were observed between groups, indicating that carnosine supplementation did not affect drinking behavior. Disease progression was evaluated based on body weight loss, as previously described for the **SOD1^G93A^** rat model [5] and according to the scoring system already proposed [23]. In this model, progressive body weight loss is a well-established, objective indicator of disease progression: peak body weight is attained at approximately 110–120 days of age (113.6 ± 4.8 days), preceding the onset of overt motor deficits (muscle weakness onset, 125.2 ± 7.4 days) by approximately 13 days, and disease stages defined by body weight and functional measures correlate with the loss of spinal motor neurons, which reaches approximately 50% at muscle weakness onset and 25% at end-stage disease [23]. Body weight loss in this model should therefore be interpreted primarily as a consequence of disease progression, reflecting motor neuron loss and consequent muscle weakness, although advanced disease and associated functional impairment may also reduce food and water intake and thereby contribute to weight loss. To minimize animal suffering, rats were euthanized when they reached a 20% reduction in body weight relative to the maximum weight recorded during the observation period (130–134 days of age). Following euthanasia, tissues were collected and stored at −80 °C until further analysis. No blinding was performed at any stage of the experiment, and randomization was not used. To minimize potential confounders, all groups were handled and experimental procedures were performed at the same time of day. Animals were housed in cages according to group (WT, WT_CAR, ALS, and ALS_CAR), with no more than 5 animals per cage. Sample sizes were determined based on reproducibility data from previous studies conducted by our group, rather than a formal a priori power calculation. One WT untreated rat cerebellum was lost during tissue dissection. The experimental groups were as follows:

WT: Animals negative for the SOD1^G93A^ gene. Did not receive treatment.

WT_CAR: Animals negative for the SOD1^G93A^ gene. The drinking bottles contained carnosine solution at 1 mg/mL ad libitum.

ALS: Animals positive for the SOD1^G93A^ gene. Did not receive treatment.

ALS_CAR: Animals positive for the SOD1^G93A^ gene. The drinking bottles contained carnosine solution at 1 mg/mL ad libitum.

### 2.2. Mass Spectrometry-Based Proteomic Analysis of Cerebellar Tissue

Protein extraction and digestion. Protein extraction and precipitation were performed from 20 mg of cerebellar tissue. The method used consisted of acetone precipitation, with acetone previously chilled overnight at −20 °C. The samples were weighed, and 300 µL of lysis solution was added: RIPA buffer (50 mM Tris (pH 7.4), 150 mM NaCl, 1% Igepal CA-630, 50% SDC, 0.1% SDS, and cOmplete™ protease inhibitor cocktail, Roche, Basel, Switzerland). The samples were manually homogenized using a tissue homogenizer, which was kept on ice throughout the entire process. Subsequently, the samples were transferred to 1.5 mL microtubes, sonicated three times for 30 s with one-minute intervals, and incubated on ice for 30 min. After incubation, the samples were centrifuged at 4 °C and 14,000 rpm for 15 min to remove cellular or tissue debris, and the supernatant was transferred to 2 mL microtubes. The samples and acetone were then kept on dry ice, and a volume of cold acetone four times greater than the volume of solution in each microtube was added as previously reported in the literature [24,25]. The samples were vortexed and kept overnight at −20 °C for protein precipitation. On the following day, centrifugation was performed at 14,000 rpm and 4 °C for 15 min. The supernatant was carefully discarded, while the pellet was dried at room temperature, avoiding complete drying. Finally, the proteins were resuspended in a solution containing 100 mM ammonium bicarbonate, 8 M urea, and deionized water using an ultrasonic bath.

The concentration of proteins in the lysate samples was measured by the bicinchoninic acid assay (BCA). Aliquots of 10 µg were prepared for subsequent protein cleanup and digestion, following previously described protocols, which were modified by passivating the filter units with 0.1% Tween-20 (v:v) instead of 5%. The peptides were dried and resuspended in a solution containing 0.1% formic acid until reaching a final concentration of 0.1 µg/µL and were analyzed by nanoLC-MS/MS.

### 2.3. LC-MS/MS Measurements

Peptide samples were analyzed using an Orbitrap Ascend Tribrid mass spectrometer (Thermo Fisher Scientific, Bremen, Germany) coupled to a Vanquish Neo nano-liquid chromatography (nano-LC) system (Thermo Fisher Scientific) equipped with a Nanospray Flex™ Ion Source. The LC–DIA–MS/MS method was adapted from previously described Orbitrap Ascend and DIA-based workflows [26,27]. Peptides were separated on a reversed-phase DNV PepMap™ Neo analytical column (150 mm × 75 μm inner diameter, 2 μm particle size) in combination with an Acclaim™ PepMap™ 100 C18 trap column (20 mm × 75 μm, 3 μm), both from Thermo Fisher Scientific. Mobile phase A consisted of 0.1% formic acid in water, and mobile phase B consisted of 0.1% formic acid in 80% acetonitrile. Peptides were eluted using a linear gradient of 3–60% solvent B over 117 min at a flow rate of 300 nL/min. The spray voltage was set to 2.2 kV, and the ion transfer tube temperature was maintained at 280 °C.

Mass spectra were acquired using a data-independent acquisition (DIA) strategy. Full MS (MS1) scans were acquired at a resolution of 60,000 over a mass range of *m*/*z* 400–900 with an automatic gain control (AGC) target of 300% and automatic maximum injection time. DIA MS/MS scans were acquired at a resolution of 15,000 using sequential 10 *m*/*z* isolation windows covering the full precursor mass range, with an AGC target of 400%, a maximum injection time of 27 ms, and a normalized collision energy of 30%.

### 2.4. Data Processing and Statistical Analysis

DIA data were processed using DIA-NN (v2.2.5) for peptide and protein identification and label-free quantification [28]. Peptide sequence searches were performed against the UniProt reference proteome of *Rattus norvegicus*, containing 61,540 protein entries and accessed in July 2026. An in silico-predicted spectral library was generated from the reference protein database, including predicted fragment-ion spectra and retention times. Searches allowed a maximum of two missed cleavages and considered peptides ranging from 7 to 30 amino acids. Carbamidomethylation of cysteine was defined as a fixed modification, whereas methionine oxidation and protein N-terminal methionine excision were included as variable modifications, with a maximum of two variable modifications per peptide. Mass tolerances were automatically optimized by DIA-NN, resulting in a final optimized mass accuracy of 7 ppm. The match-between-runs option was enabled to improve data completeness across samples, and protein quantities were estimated using the QuantUMS high-precision quantification strategy [29]. Precursor- and protein-level identifications were controlled at a false discovery rate (FDR) of 1%. Downstream analyses were conducted in R (v4.1.3). The DIA-NN precursor report and protein-group abundance matrix were imported using the arrow and base R packages, respectively. Protein groups were retained only when they fulfilled all four DIA-NN confidence criteria: library precursor q-value (Lib.Q.Value) < 0.01, library protein-group q-value (Lib.PG.Q.Value) < 0.01, precursor q-value (Q.Value) < 0.01, and protein-group q-value (PG.Q.Value) < 0.01. Protein groups were further required to contain more than one identified peptide sequence and at least one proteotypic peptide sequence. Protein groups annotated as contaminants were excluded. To retain proteins consistently quantified across the experimental conditions, proteins were required to have fewer than three missing abundance values within each experimental group. Missing values were not imputed. Protein abundance values were log_2_-transformed and normalized across samples by quantile normalization using the “normalizeBetweenArrays” function from the limma package. Differential protein abundance was assessed using the limma Bioconductor package (v3.50.3) [30]. Differential protein abundance was assessed by fitting a linear model to the normalized protein-abundance matrix, with separate coefficients for the ALS, ALS_CAR, WT, and WT_CAR groups. Three predefined pairwise contrasts were evaluated: ALS versus WT, ALS_CAR versus ALS, and WT_CAR versus WT. Contrast-specific statistics were calculated using empirical Bayes moderation of the protein-wise standard errors. Resulting *p*-values were adjusted for multiple testing using the Benjamini–Hochberg procedure. Proteins were considered differentially abundant when they presented an adjusted *p*-value < 0.05 and an absolute log_2_ fold change > 0.5. Effect sizes are reported as log_2_ fold changes derived from the limma linear model; 95% confidence intervals for individual fold changes were not computed, as significance was assessed via moderated t-statistics and Benjamini–Hochberg-adjusted *p*-values. Positive and negative log_2_ fold changes were classified as increased and decreased abundance, respectively, relative to the denominator group of each contrast. Reactome pathway enrichment analysis was conducted separately for proteins with increased and decreased abundance in each comparison. Gene symbols were converted to Entrez Gene identifiers using the org.Rn.eg.db annotation package (version 3.20.0) for Rattus norvegicus. Enrichment analyses were performed with ReactomePA (version 1.52.0) [31] through the compareCluster framework implemented in clusterProfiler (version 4.16.0) [32]. Only protein sets containing at least three successfully mapped Entrez Gene identifiers were analyzed. Reactome pathways and Gene Ontology Biological Processes with Benjamini–Hochberg adjusted *p*-values < 0.05 were retained for visualization, as indicated in the legends of the figures, and the five highest-ranking pathways for each direction of regulation were displayed as dot plots. Dot size represented the number of proteins associated with each pathway, whereas dot color represented enrichment significance. Protein–protein interaction (PPI) networks were generated using the STRINGdb package (version 2.20.0) [33] based on interaction data retrieved from the STRING database v12.0. Protein identifiers were mapped to STRING identifiers using the *Rattus norvegicus* reference proteome (NCBI taxonomy ID: 10116). Networks were constructed using the differentially abundant proteins identified in each comparison, considering both experimentally validated and predicted functional associations available in STRING. Unless otherwise specified, the default STRING confidence score threshold (combined score ≥ 0.4, medium confidence) was used. Network visualization was performed with the functions implemented in the STRINGdb package. Data Availability (see Supplementary Information).

## 3. Results

**Figure 1:**
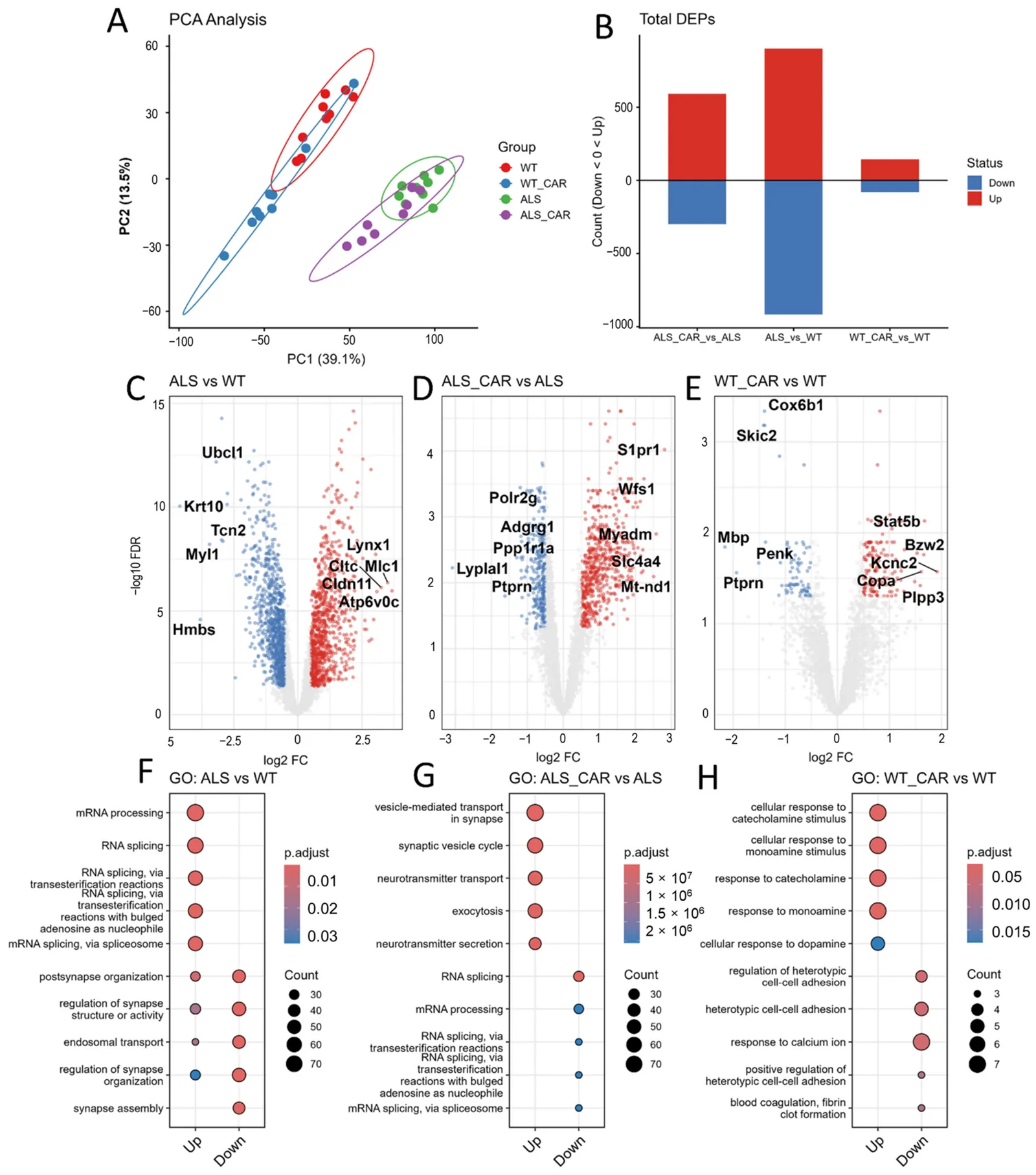
**Carnosine induces substantially greater proteome remodeling in ALS rats than in wild-type rats.** (**A**) Principal component analysis of the protein-abundance matrix showing the distribution of samples from the ALS (n = 8), ALS_CAR (n = 9), WT (n = 8), and WT_CAR (n = 9) groups. The percentages shown on the axes indicate the proportion of total variance explained by the first two principal components, and each point represents one biological sample. (**B**) The bar plot summarizes the number of differentially abundant proteins identified in each comparison, with proteins of increased and decreased abundance shown above and below zero, respectively. (**C**–**E**) Volcano plots show differential protein abundance for ALS versus WT, ALS_CAR versus ALS, and WT_CAR versus WT, respectively. Statistical comparisons were performed using limma with empirical Bayes moderation. Proteins were considered differentially abundant when they presented a Benjamini–Hochberg-adjusted *p*-value < 0.05 and an absolute log_2_ fold change > 0.5. Proteins with increased and decreased abundance are shown in red and blue, respectively, whereas non-significant proteins are shown in gray. The most strongly regulated proteins in each direction are labeled. (**F**–**H**) Gene Ontology Biological Process enrichment analyses were performed separately for proteins with increased and decreased abundance in each comparison. The five highest-ranking significantly enriched pathways are displayed as dot plots, with dot size indicating the number of proteins associated with each pathway and dot color representing the adjusted *p*-value.

Quantitative proteomic analysis was performed on the four experimental groups (WT, WT_CAR, ALS, and ALS_CAR) to investigate the effects of carnosine treatment in an ALS rat model. Principal component analysis of the quantified proteome showed that PC1 (39.1% of variance) segregated samples almost exclusively by genotype: WT and WT_CAR clustered together, while ALS and ALS_CAR formed a separate, non-overlapping cluster (Figure 1A). Carnosine treatment did not shift ALS_CAR samples toward the WT/WT_CAR cluster; instead, ALS_CAR animals occupied a distinct region of PC1–PC2 space, indicating that carnosine reorganizes the ALS cerebellar proteome without restoring a wild-type-like state. Differential expression analysis (limma, FDR < 0.05) supported this interpretation. The ALS genotype alone yielded 1817 differentially expressed proteins (DEPs) relative to WT (917 down-regulated, 900 up-regulated; Figure 1B,C). Carnosine altered 890 proteins in ALS animals relative to untreated ALS (298 down, 592 up; Figure 1B,D), but only 224 proteins in WT animals relative to untreated WT (80 down, 144 up; Figure 1B,E)—a four-fold difference in the number of carnosine-responsive proteins between genotypes. This disparity indicates that the cerebellar proteomic response to carnosine is strongly potentiated by the ALS disease context rather than reflecting a fixed, genotype-independent pharmacological effect.

Gene Ontology Biological Process enrichment analysis revealed distinct functional patterns across the three comparisons. In ALS relative to WT, proteins with increased abundance were predominantly associated with mRNA processing and RNA-splicing pathways, including spliceosome-mediated transesterification reactions (shown in Figure 1F). Conversely, proteins with decreased abundance were enriched in processes related to postsynaptic organization, synapse assembly, regulation of synaptic structure and activity, and endosomal transport (Figure 1F). In ALS_CAR relative to ALS, proteins with increased abundance were enriched in vesicle-mediated transport in synapses, the synaptic vesicle cycle, neurotransmitter transport and secretion, and exocytosis. Proteins with decreased abundance were primarily associated with RNA splicing and mRNA processing, including spliceosome-dependent reactions (Figure 1G). In WT_CAR relative to WT, the proteomic response was characterized by enrichment of catecholamine-, monoamine-, and dopamine-responsive processes among proteins with increased abundance, whereas proteins with decreased abundance were associated with heterotypic cell–cell adhesion, calcium-ion responses, and fibrin clot formation (Figure 1H). Throughout the Section 3, individual proteins are highlighted as representative members of these statistically enriched functional modules rather than as an a priori curated list of hypothesized disease-driving or “key regulator” genes; protein selection was data-driven, based on differential abundance testing (limma), Gene Ontology enrichment, hierarchical clustering, and STRING protein–protein interaction network analysis, and the specific proteins discussed are intended to illustrate broader pathway-level trends rather than to imply individual prioritization.

Among the 469 proteins significantly altered in both the ALS-vs-WT and ALS_CAR-vs-ALS comparisons, only 106 (22.6%) changed in the opposite direction with treatment—i.e., moved toward the WT profile—while the remaining 363 (77.4%) changed in the same direction as the disease-associated alteration, indicating exacerbation rather than correction. This asymmetry was most pronounced among proteins up-regulated in ALS: of 303 such proteins also modulated by carnosine, 268 (88.4%) were further increased in ALS_CAR, and only 35 (11.6%) were reduced toward the WT baseline. Among the 166 proteins down-regulated in ALS, the effect was more balanced, with 95 (57.2%) further decreased by carnosine and 71 (42.8%) partially recovered.

Hierarchical clustering of the 890 carnosine-responsive proteins in ALS animals resolved six clusters with distinct relationships to the underlying disease signature (shown in Supplementary Figure S1). The largest cluster (Cluster 1, n = 400, up-regulated by carnosine) was dominated by proteins already elevated in ALS (67% overlap with the ALS-vs-WT signature), none of which reversed direction, consistent with pure amplification; this cluster included membrane ion pumps and transporters (*Atp2a2*, *Atp2a3*, *Atp2b4*, *Atp4a*, *Atp6v0a1*, and *Atp6v0c*), a mitochondrial respiratory chain subunit (*Mt-nd1*), an antioxidant enzyme (*Mgst3*), and synaptic vesicle trafficking components (*Scamp5* and *Cltc*). By contrast, Clusters 2 and 3 (n = 159 and 33, both up-regulated) contained 38 and 33 proteins, respectively, that reversed an ALS-associated decrease, comprising ion channels and transporters (*Cacna1a*, *Kcnc1*, *Kcnc3*, *Slc12a5*, *Slc4a10*, *Slc20a2*, and *Gabbr2*), SNARE and synaptic vesicle components (*Stx1b*, *Stx4*, *Syt1*, *Syt3*, *Vti1a*, and *Vti1b*), and autophagy-related proteins (*Atg9a* and *Mtmr2*). Clusters 5 and 6 (n = 55 and 44, down-regulated by carnosine) were enriched for RNA polymerase II subunits (*Polr2g* and *Polr2h*), pre-mRNA splicing factors (*Rbm17*, *Snrpa1*, *Sf3b4*, *Hnrnpa0*, and *Rbfox3*), and the proteasome subunits *Psma7* and *Psmd10*, the majority of which were reduced from an ALS-associated elevation, consistent with partial normalization of a splicing/proteostasis stress signature. Cluster 4 (n = 199, down-regulated by carnosine) largely reflected further suppression of proteins already reduced in ALS (47.7% overlap, 0% reversal). Proteasome-related proteins were distributed across Clusters 1, 4, and 6, indicating heterogeneous responses (proteins modulated in each limma comparison are available in the Supplementary Information).

Gene Ontology enrichment mirrored this pattern: proteins up-regulated by carnosine in ALS animals were enriched for vesicle-mediated transport in synapse, synaptic vesicle cycle, neurotransmitter transport/secretion, and exocytosis (all FDR < 2 × 10^−6^), whereas down-regulated proteins were enriched for RNA splicing and mRNA processing terms (Figure 1).

**Figure 2:**
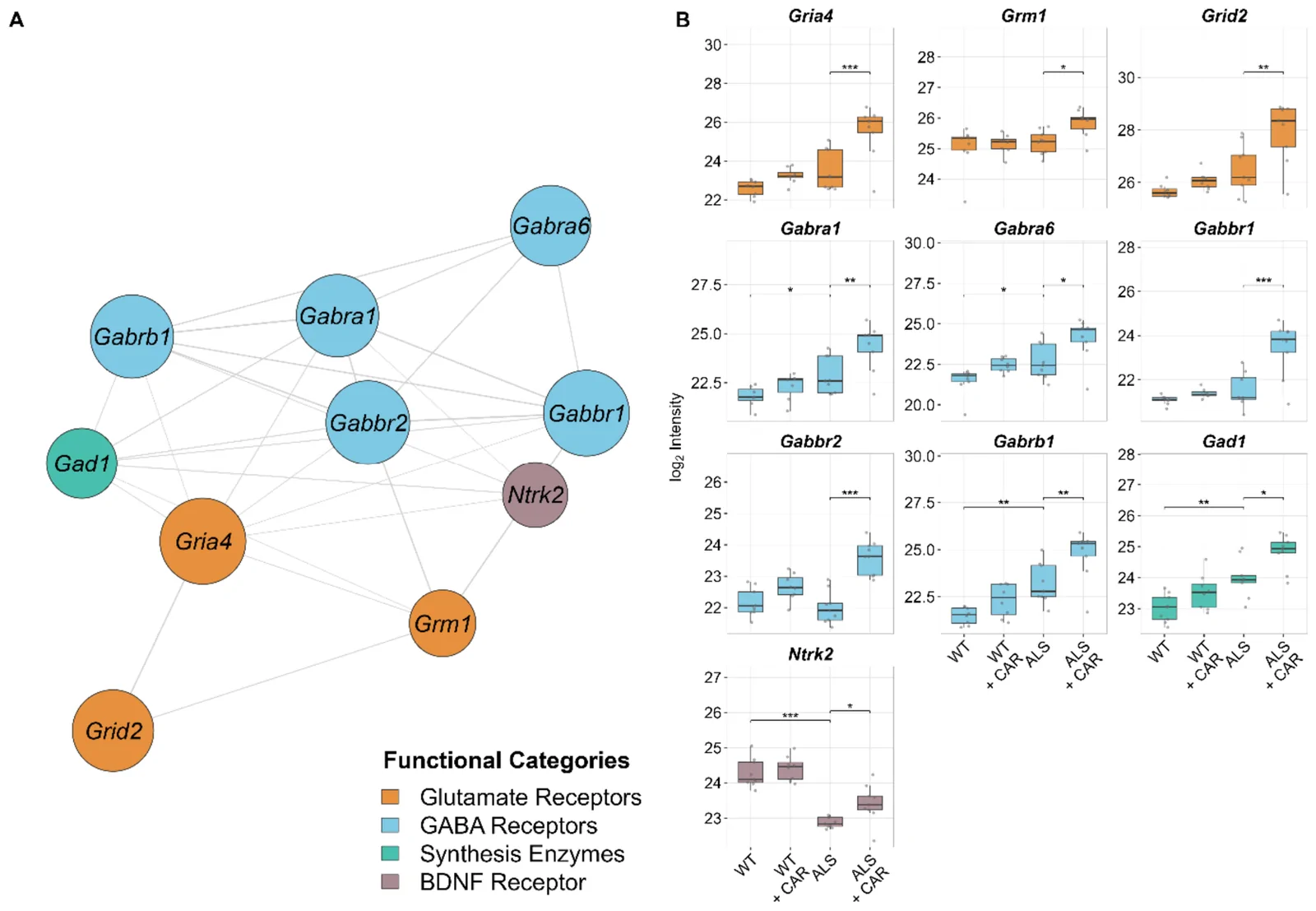
**Carnosine selectively reshapes proteins involved in excitatory and inhibitory neurotransmission in ALS rats.** (**A**) Protein–protein interaction network of synaptic proteins significantly regulated in ALS_CAR compared with untreated ALS animals. Proteins were considered differentially abundant when the Benjamini–Hochberg-adjusted *p*-value was <0.05 and |log_2_ fold change| was >0.5. Synaptic proteins were selected based on significantly enriched Gene Ontology Biological Process and Reactome terms, followed by manual curation into functional categories related to excitatory and inhibitory neurotransmission. Interactions were retrieved from STRING v12.0 for *Rattus norvegicus* using a minimum combined confidence score of 0.4. Nodes represent proteins, and edges represent known or predicted functional associations. Node colors indicate functional categories, including glutamate receptors, GABA receptors, neurotransmitter synthesis enzymes, and the BDNF receptor. Node size is proportional to the absolute log_2_ fold change in the ALS_CAR versus ALS comparison. (**B**) Boxplots showing normalized log_2_ protein intensities across WT, WT_CAR, ALS, and ALS_CAR animals. Boxes indicate the interquartile range, center lines indicate the median, and individual points represent biological replicates. Statistical comparisons were performed using limma with empirical Bayes moderation, and *p*-values were adjusted using the Benjamini–Hochberg procedure. Significance annotations are shown for WT_CAR versus WT, ALS versus WT, and ALS_CAR versus ALS. Only significant comparisons are annotated: * adjusted p* < 0.05 (*), <0.01 (**), and <0.001 (***).

Given the enrichment of synaptic and vesicle-trafficking terms among carnosine-responsive proteins, we examined ionotropic and metabotropic glutamate and GABA receptor subunits directly, as well as other proteins involved in neurotransmission (Figure 2). Several receptor subunits that were not significantly altered in ALS relative to WT became significantly up-regulated by carnosine specifically in the ALS background, including the AMPA receptor subunit *Gria4* (log2FC = 2.06), the glutamate metabotropic receptor 1 *Grm1* (log2FC = 0.62), the delta-type glutamate receptor *Grid2* (log2FC = 1.46), and the GABA-B receptor subunits *Gabbr1* (log2FC = 1.92) and *Gabbr2* (log2FC = 1.60). Other subunits already elevated in ALS relative to WT (*Gabra1*, log2FC = 1.04; *Gabra6*, log2FC = 1.20; *Gabrb1*, log2FC = 1.70, and *Gad1*, log2FC = 0.97) were further increased by carnosine (log2FC = 1.52, 1.41, 1.56, and 0.83 respectively). The BDNF receptor *Ntrk2*/TrkB, one of the most strongly down-regulated proteins in ALS relative to WT (log2FC = −1.41), was partially restored by carnosine (log2FC = +0.55), representing one of the clearest examples of treatment-associated reversal of an ALS-associated deficit.

**Figure 3:**
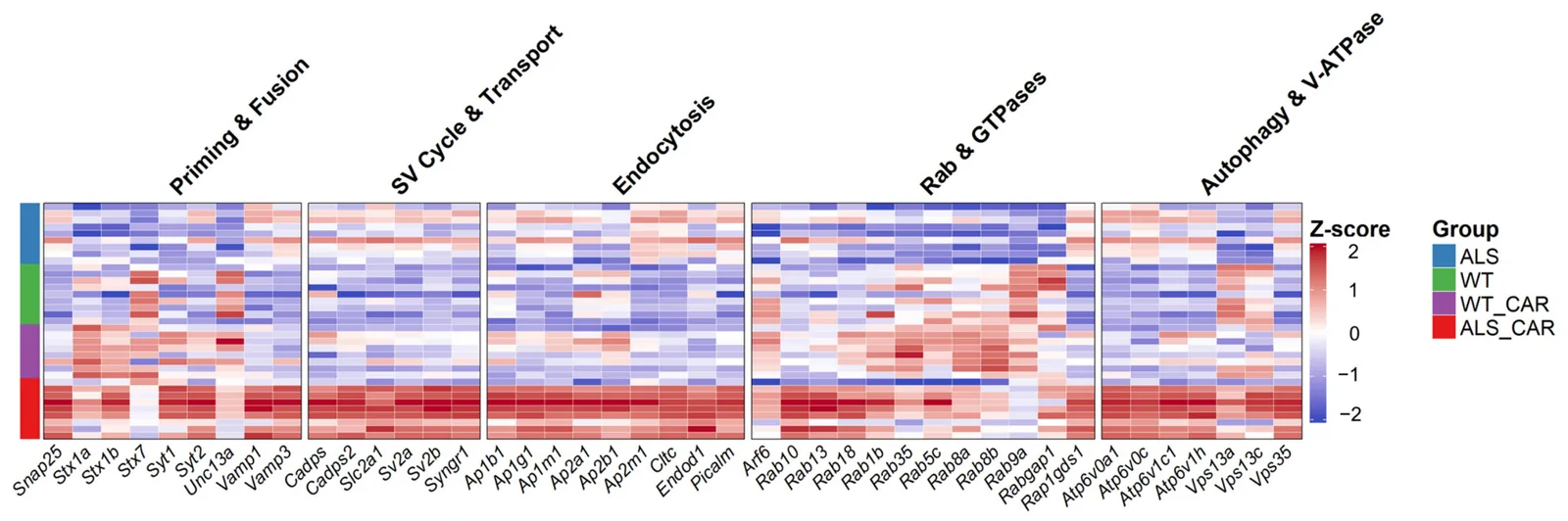
**Synaptic protein abundance profiles across experimental groups.** Heatmap showing the standardized relative abundance of selected proteins associated with major synaptic processes in cerebellar samples from WT, WT_CAR, ALS, and ALS_CAR animals. Proteins were selected from those showing differential abundance in ALS_CAR versus ALS animals, defined by a Benjamini–Hochberg-adjusted *p*-value < 0.05 and an absolute log_2_ fold change > 0.5. Statistical comparisons were performed using limma with empirical Bayes moderation. Synaptic proteins were identified based on significantly enriched Gene Ontology Biological Process and Reactome terms and were subsequently manually curated into functional categories related to synaptic vesicle priming and fusion, synaptic vesicle cycling and transport, endocytosis, Rab proteins and other GTPases, and autophagy and V-ATPase function. Rows represent proteins, and columns represent individual biological samples. For each protein, normalized log_2_ abundance values were standardized across all samples as row-wise Z-scores. Red and blue indicate relatively higher and lower abundance, respectively. The upper annotation bar indicates the experimental group of each sample, whereas the annotation bar on the left indicates the functional category assigned to each protein.

Consistent with this, a curated panel of synaptic vesicle cycle, priming/fusion, endocytosis, Rab GTPase, and autophagy/V-ATPase proteins showed a graded increase across WT < WT_CAR < ALS < ALS_CAR groups, with ALS_CAR values frequently exceeding those of WT (Figure 3; a table listing proteins significantly modulated in the ALS_CAR versus WT comparison is provided in Supplementary Information).

**Figure 4:**
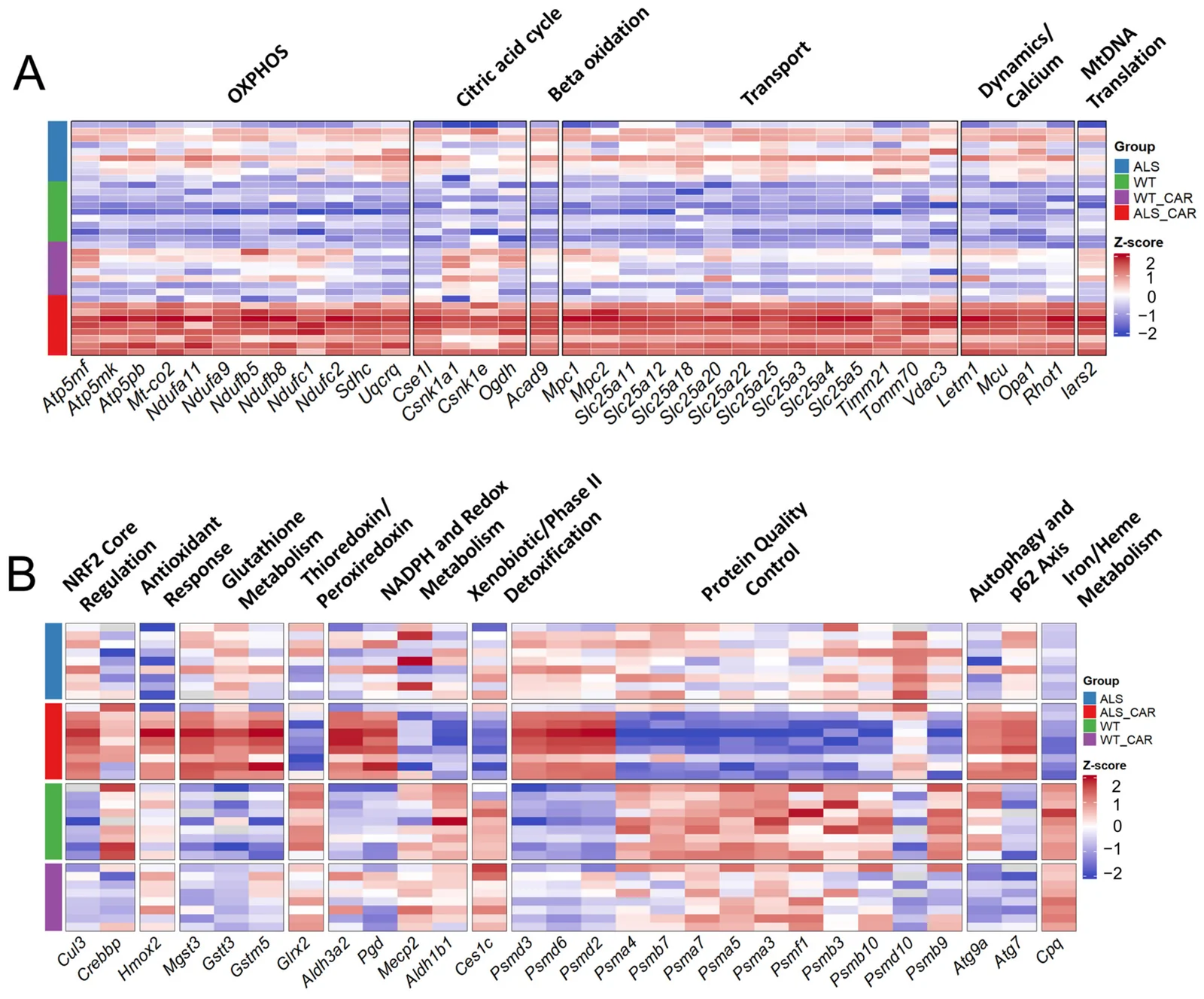
**Carnosine supplementation induces coordinated remodeling of mitochondrial and redox-associated protein networks in the ALS cerebellum.** Heatmaps show the relative abundance profiles of proteins significantly altered in ALS_CAR compared with untreated ALS animals. Differentially abundant proteins were defined by a Benjamini–Hochberg-adjusted *p*-value < 0.05 and an absolute log2 fold change > 0.5 using a limma model. Although protein selection was based on the ALS_CAR versus ALS comparison, abundance profiles are shown across all experimental groups: WT, WT_CAR, ALS, and ALS_CAR. Protein abundances were standardized across samples and are represented as row Z-scores, with blue and red indicating lower and higher relative abundance, respectively. Samples are displayed in rows and proteins in columns. Proteins were assigned to functional categories based on Gene Ontology Biological Process and Reactome annotations, followed by manual curation. (**A**) Mitochondrial proteins grouped according to functional categories, including oxidative phosphorylation, the citric acid cycle, beta-oxidation, mitochondrial transport, mitochondrial dynamics and calcium homeostasis, and mitochondrial DNA maintenance and translation. (**B**) Proteins associated with NRF2 signaling and redox homeostasis, grouped into NRF2 core regulation, antioxidant response, glutathione metabolism, thioredoxin/peroxiredoxin systems, NADPH and redox metabolism, xenobiotic and phase II detoxification, protein quality control, autophagy and the p62 axis, and iron/heme metabolism. The lateral annotation indicates the experimental group of each sample, whereas the upper annotations indicate the functional category assigned to each protein.

Pathway-level analysis of carnosine-responsive proteins in ALS animals revealed coordinated up-regulation of oxidative phosphorylation complex subunits, tricarboxylic acid cycle enzymes, beta-oxidation, mitochondrial transporters/carriers, and mitochondrial dynamics/calcium-handling proteins (Figure 4A), together with NRF2-associated antioxidant response, glutathione metabolism, and NADPH/redox metabolism proteins (Figure 4B).

In nearly all of these categories, ALS_CAR values exceeded not only untreated ALS but also WT, indicating that carnosine drives cerebellar mitochondrial and antioxidant capacity beyond baseline physiological levels rather than simply restoring an ALS-associated deficit. In contrast, the protein quality control module—comprising 20S/26S proteasome subunits (*Psma3*, *Psma4*, *Psma5*, *Psma7*, *Psmb3*, *Psmb7*, *Psmb9*, *Psmb10*, *Psmd2*, *Psmd3*, *Psmd6*, *Psmd10*, and *Psmf1*)—showed the opposite pattern, with pronounced down-regulation in ALS_CAR relative to both ALS and WT_CAR (Figure 4B); of these, only *Psma7* and *Psmd10* correspond to the splicing/proteostasis-normalization signature of Clusters 5–6; most of the remaining down-regulated subunits fall within Cluster 4 (general further suppression), and *Psmd2*, *Psmd3*, and *Psmd6* instead increased (Supplementary Figure S1). At the individual protein level, this included up-regulated subunits of Complex I (*Ndufb8*, *Ndufa9*, *Ndufc2*, *Ndufb6*, *Ndufa11*, and *Ndufb2*), Complex II (*Sdhc*), Complex III (*Uqcrq*), Complex IV (*Mt-Co2*), and ATP synthase (*Atp5pb*), together with the mitochondrial matrix protease *Afg3l2*, whereas canonical ROS-scavenging enzymes (e.g., *Sod1*, *Sod2*, *Gpx*, *Prdx*, and *Cat*) and *AIFM1* did not reach individual significance. At the pathway level, gene set enrichment analysis of NRF2-associated and oxidative-stress-related terms (Supplementary Figure S3) nonetheless revealed coordinated, significant enrichment in the ALS_CAR versus ALS comparison, with detoxification- and hydrogen-peroxide-related processes enriched among up-regulated proteins and terms reflecting direct nuclear NFE2L2/KEAP1 signaling enriched among down-regulated proteins, indicating that carnosine’s redox-related effect in this model is distributed across many proteins of modest individual effect size rather than concentrated in a single canonical ROS-scavenging enzyme. A focused enrichment analysis of mitochondria-related biological processes across the three experimental comparisons (Supplementary Figure S2) provided further pathway-level support for the individual mitochondrial findings described above.

**Figure 5:**
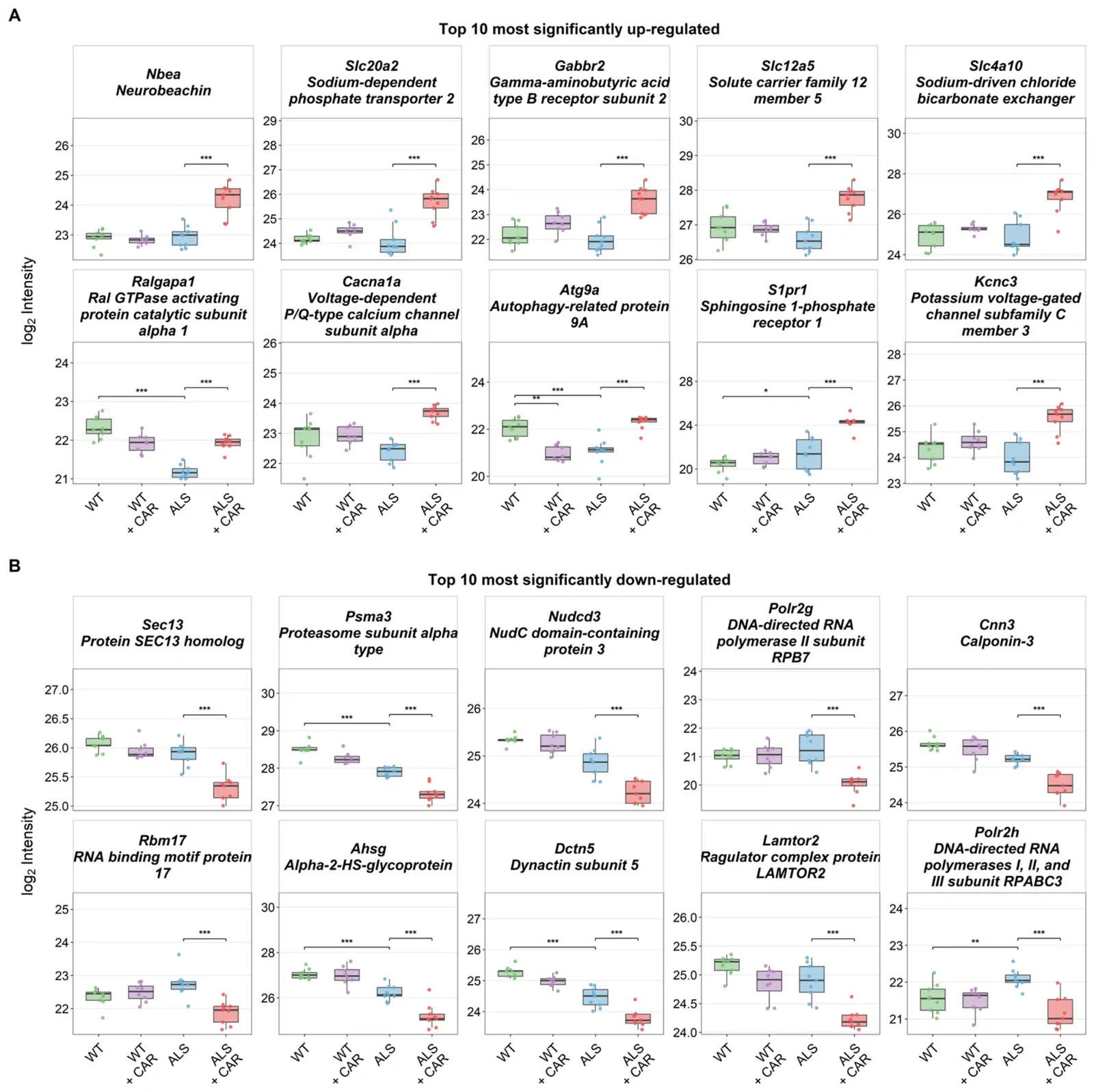
**Carnosine up-regulates proteins involved in neurotransmission while attenuating the expression of proteins involved in RNA processing and gene expression in ALS rats.** (**A**) Boxplots showing the ten most significantly up-regulated proteins in ALS_CAR compared with untreated ALS animals. (**B**) Boxplots showing the ten most significantly down-regulated proteins in the same comparison. Proteins were selected based on the ALS_CAR versus ALS contrast using limma with empirical Bayes moderation and ranked according to the Benjamini–Hochberg-adjusted *p*-value after applying the significance criteria of adjusted *p* < 0.05 and |log2 fold change| > 0.5. Protein abundances are displayed as normalized log_2_ intensities for all experimental groups (WT, WT_CAR, ALS, and ALS_CAR). Boxes represent the interquartile range, center lines indicate the median, whiskers extend to 1.5× the interquartile range, and individual points represent biological replicates. Statistical comparisons were performed using limma with empirical Bayes moderation, and adjusted *p*-values are indicated for WT_CAR versus WT, ALS versus WT, and ALS_CAR versus ALS. Only significant comparisons are annotated: * adjusted p* < 0.05 (*), <0.01 (**), and <0.001 (***).

Among the ten most significantly up-regulated proteins (Figure 5A), several were not significantly altered in ALS relative to WT and emerged as carnosine-specific responses restricted to the ALS genotype, including *Nbea*, *Slc20a2*, *Gabbr2*, *Slc12a5*, *Slc4a10*, *Atg9a*, *Mtmr6*, and *S1pr1*, whereas *Ralgapa1* and *Cacna1a* showed significant changes across multiple pairwise comparisons. Among the ten most significantly down-regulated proteins in ALS_CAR relative to ALS (Figure 5B), several showed a stepwise decline across WT→WT_CAR→ALS→ALS_CAR (*Sec13*, *Psma3*, *Nudcd3*, *Cnn3*, *Ahsg*, *Dctn5*, and *Lamtor2*), while others (*Polr2g*, *Rbm17*, and *Polr2h*) declined specifically with carnosine treatment in the ALS background, without a corresponding difference between untreated ALS and WT.

To distinguish disease-context-dependent effects of carnosine from a direct, genotype-independent pharmacological action, we compared the ALS_CAR-vs-ALS and WT_CAR-vs-WT signatures. Of the 224 proteins altered by carnosine in WT animals, 72 (32.1%) were also altered by carnosine in ALS animals, and of these, 63 (87.5%) changed in the same direction in both genotypes, indicating a shared, genotype-independent component of the carnosine response. However, this shared core represented only 8.1% of the total carnosine-responsive proteome in ALS animals (72/890), indicating that the large majority of carnosine-induced proteomic remodeling in the ALS cerebellum is disease-context-specific rather than a generic pharmacological signature.

## 4. Discussion

This study characterized the cerebellar proteomic response to carnosine supplementation in an ALS-model rat and compared it directly to the proteomic signature of disease itself. Two findings define the overall picture. First, the ALS genotype produced extensive, genome-wide proteomic remodeling of the cerebellum—affecting nearly 1800 proteins—that dominated the principal component structure of the dataset, consistent with the cerebellum being an actively affected, rather than spared, region in this model. Second, and less expected, carnosine treatment did not reposition the ALS proteome toward the wild-type state. Instead, it produced a signature unique to the ALS_CAR group, built predominantly on amplification of pathways already altered by disease rather than their correction. This distinction between amplification and reversal has important implications for interpreting the mechanism of action of carnosine and, more broadly, other redox-active nutraceutical interventions in preclinical models of neurodegeneration [22].

The four-fold greater number of carnosine-responsive proteins in ALS animals relative to WT (890 vs. 224) indicates that the cerebellar response to carnosine scales with the underlying disease state rather than reflecting a fixed pharmacological signature. This pattern is compatible with the established biochemistry of carnosine, a histidine-containing dipeptide with antioxidant, anti-glycation, pH-buffering, and transition-metal-chelating properties whose activity is expected to be most consequential under conditions of elevated oxidative and metabolic stress—precisely the intracellular environment associated with ALS pathophysiology, including mitochondrial dysfunction, impaired calcium handling, and glutamate-mediated excitotoxicity. The pathway-level data support this interpretation: oxidative phosphorylation subunits, tricarboxylic acid cycle enzymes, mitochondrial calcium-handling proteins, and NRF2-associated antioxidant and glutathione-metabolism proteins were coordinately elevated in ALS_CAR animals, frequently exceeding wild-type levels (Figure 4). Notably, this redox signature was not accompanied by significant changes in individual canonical ROS-scavenging enzymes or AIFM1-related apoptotic markers; rather, pathway-level analysis indicated a coordinated shift distributed across many proteins of modest effect size, coupled to a reduction in direct nuclear NFE2L2/KEAP1 signaling (Supplementary Figures S2 and S3), suggesting that carnosine’s antioxidant effect in this model operates primarily through redistribution of redox-related protein networks rather than induction of a single dominant detoxification enzyme. A parallel pattern was observed for synaptic vesicle cycling, exocytosis, and neurotransmitter transport machinery (Figure 1 and Figure 2), which were likewise potentiated beyond both the ALS and WT baselines.

Whether this hypercompensation is adaptive or maladaptive cannot be resolved from proteomic abundance alone. On one hand, boosting mitochondrial bioenergetic capacity, antioxidant buffering, and synaptic vesicle turnover beyond physiological levels could represent a therapeutically desirable expansion of cellular reserve capacity in a tissue under chronic metabolic strain. On the other hand, sustained overexpression of ion pumps, transporters, and vesicle-trafficking machinery carries its own energetic and proteostatic cost and could, in principle, represent a new non-physiological steady state rather than a return to healthy function. Distinguishing between these possibilities will require functional correlates—mitochondrial respirometry, synaptic electrophysiology, calcium imaging, and behavioral/motor outcome measures—that were outside the scope of the present proteomic dataset. Nonetheless, the magnitude of change observed in synaptic vesicle cycling, priming/fusion, endocytosis, and Rab-GTPase-associated proteins (Figure 3) is consistent in direction and scale with alterations previously linked to demonstrable structural and functional synaptic consequences in the C9orf72-ALS/FTD cerebellum [34], and with recent clinical evidence that cerebrospinal fluid levels of vesicle-associated and presynaptic proteins, including VAMP2 and SNAP25, track relative disease stage and survival in human ALS [35], supporting the plausibility that the vesicle-trafficking changes reported here reflect functionally consequential synaptic remodeling. Direct confirmation via patch-clamp electrophysiology and/or synaptic vesicle release assays (e.g., FM dye imaging) in cerebellar slices remains an important direction for future work. Additionally, effect sizes are reported as log2 fold changes without confidence intervals, which should be considered when interpreting the magnitude of individual protein changes.

Within this broader pattern of amplification, a subset of proteins showed genuine reversal of the ALS-associated signature. The most notable was the BDNF receptor *Ntrk2*/TrkB, which was substantially reduced in ALS relative to WT and partially restored by carnosine. Loss of BDNF/TrkB signaling is a well-documented feature of motor neuron degeneration and has been linked to impaired trophic support and synaptic maintenance [36]. Its partial normalization here—together with the up-regulation of GABA-B and ionotropic/metabotropic glutamate receptor subunits (Figure 2)—raises the possibility that carnosine acts, at least in part, through modulation of neurotrophic and synaptic receptor signaling in addition to its established antioxidant activity. We further examined whether this increase in receptor subunit abundance was accompanied by changes in well-characterized auxiliary or scaffolding proteins that stabilize glutamatergic and GABAergic receptors at the synapse (e.g., the postsynaptic scaffold *Dlg4*/PSD-95, *Shank1–3*, the AMPA receptor auxiliary subunit *Cacng2*/stargazin, and the synaptic adhesion molecules *Nlgn1–3*/neuroligins and *Nrxn1–3*/neurexins); none of these were among the significantly modulated proteins in the ALS_CAR versus ALS comparison, indicating that the increase in receptor subunit abundance shown in Figure 2 was not accompanied by a detectable parallel change in this specific set of stabilizing proteins. Whether this translates into increased functional receptor stabilization at the synapse will require direct assessment of receptor trafficking and surface expression, for example, by surface biotinylation or super-resolution imaging. The cerebellar localization of this effect is noteworthy: although ALS has traditionally been framed as a disease of upper and lower motor neurons, growing evidence implicates cerebellar [37] and extra-motor circuits in ALS and in the ALS-frontotemporal dementia spectrum, and the present data suggest that this region is both transcriptionally/proteomically responsive to disease and pharmacologically modifiable.

A second, smaller subset of proteins showed reversal in the opposite direction: RNA polymerase II subunits, pre-mRNA splicing factors, and two 20S/26S proteasome subunits (*Psmd10* and *Psma7*) were elevated in ALS and reduced toward baseline by carnosine (Figure 5). Aberrant RNA processing and disruption of proteostasis, including impairment of the ubiquitin–proteasome system, are recognized hallmarks of ALS pathophysiology, most extensively characterized in the context of TDP-43 nuclear depletion, cytoplasmic aggregation, and cryptic splicing [38]. The reduction of this signature by carnosine could reflect attenuation of an underlying proteotoxic or splicing stress response; alternatively, it could reflect a reduction in overall proteostatic surveillance capacity. Because steady-state protein abundance does not distinguish reduced stress from reduced capacity, orthogonal assays—ubiquitinated protein load, autophagic flux, and splicing-sensitive reporter transcripts—will be required to determine which interpretation applies.

Comparison of the ALS_CAR-vs-ALS and WT_CAR-vs-WT signatures indicated that only a small fraction (72 of 890 proteins, 8.1%) of the carnosine response in ALS animals was shared with the response in WT animals, and this shared core was itself concordant in direction in 87.5% of cases. This finding supports a model in which carnosine has a modest, direction-consistent baseline pharmacological effect that is independent of disease genotype, superimposed on a much larger disease-context-specific response. From a translational standpoint, this argues against interpreting carnosine as a generic antioxidant supplement with uniform CNS effects and instead supports its evaluation specifically within conditions of elevated baseline oxidative or metabolic stress, where its proteomic impact appears to be concentrated.

Our proteomic analysis of the cerebellum is consistent with previous proteomic studies of the motor cortex in ALS patients, which likewise identified mitochondrial dysfunction as a central pathogenic feature [39]. Despite the differences between human tissue and the rat experimental model, a strong convergence in mitochondrial processes is observed between the two datasets. In that study, SWATH-MS analysis detected widespread proteomic alterations affecting pathways such as oxidative phosphorylation, glycolysis, redox regulation, and sirtuin signaling, with mitochondrial dysfunction emerging as the top canonical pathway. Although the motor cortex represents a primary site of neurodegeneration in ALS, the convergence of mitochondrial alterations observed in both datasets suggests that impaired bioenergetic homeostasis may represent a common mechanism affecting multiple brain regions. In contrast to the broader signaling and inflammatory pathways detected in the motor cortex, the alterations observed in the cerebellar mitochondrial proteome appear to be more directly related to metabolic and bioenergetic processes, including respiratory chain function and substrate metabolism. This difference may reflect regional variations in neuronal vulnerability or compensatory metabolic adaptations in the cerebellum. Nevertheless, the consistent involvement of mitochondrial pathways across studies reinforces the hypothesis that mitochondrial dysfunction and altered energy metabolism play a central role in ALS pathogenesis.

A recent quantitative proteomic study investigating the protective effects of carnosine against UV-A-induced damage in mouse skin provides interesting parallels with our findings [40]. In that study, exposure to UV-A radiation resulted in significant alterations in the skin proteome, affecting pathways related to inflammation, oxidative stress, calcium signaling, fibrogenesis, and cytoskeletal organization. Importantly, mitochondrial function and metabolism were also impaired, with altered expression of proteins involved in the respiratory chain, including cytochrome c oxidase and NADH:ubiquinone oxidoreductase. Preventive treatment with carnosine largely prevented these proteomic alterations, highlighting its protective role against oxidative and metabolic stress. Mechanistically, carnosine may exert its protective effects through multiple complementary pathways, including scavenging of reactive oxygen species, detoxification of reactive carbonyl species generated during lipid peroxidation, modulation of histaminergic pathways and stabilization of mitochondrial proteins susceptible to oxidative modification. By limiting oxidative damage and preserving respiratory chain function, carnosine may help maintain cellular bioenergetic balance under pathological conditions. Taken together, these observations support the hypothesis that the protective effects of carnosine observed in different tissues may converge on the preservation of mitochondrial integrity and metabolic homeostasis.

### Study Limitations

This study has some limitations that should be acknowledged. SOD1 mutations account for a substantial proportion of familial ALS cases; however, since familial ALS represents only ~10% of all cases, SOD1-associated disease constitutes a minority of ALS cases overall, with most cases being sporadic and SOD1-independent [2,3]. Consequently, findings from this SOD1^G93A^ overexpression model may not directly predict clinical responses in the broader ALS population. Nonetheless, SOD1^G93A^ models recapitulate several pathological features shared across ALS subtypes regardless of genetic origin—including mitochondrial dysfunction, oxidative and carbonyl stress, protein aggregation, and synaptic dysfunction—suggesting that interventions targeting these convergent downstream mechanisms, such as carnosine supplementation, may hold relevance beyond SOD1-linked disease. Validation in models more representative of sporadic ALS pathology (e.g., TDP-43-based models) or in post-mortem human tissue would strengthen confidence in translational relevance. In addition, proteomic profiling was performed on bulk cerebellar tissue, and the reported changes therefore reflect the net signature across all resident cell types without cell-type resolution; cell-type-resolved approaches, such as single-nucleus proteomics or transcriptomics, laser-capture microdissection, or immunohistochemical co-localization of key candidate proteins, will be required to determine whether the mitochondrial, synaptic, and RNA-processing signatures reported here originate predominantly from neurons, glia, or a combination of both. Furthermore, animals were compared at a single, disease-stage-matched endpoint (20% body weight loss, corresponding to end-stage disease) rather than across multiple time points, so the present dataset represents a cross-sectional snapshot rather than a proteomic trajectory of disease progression; generating such a trajectory across pre-symptomatic, onset, and end-stage time points was outside the scope of the present study and is an important direction for future longitudinal work.

## 5. Conclusions

Carnosine supplementation in this ALS-model rat did not restore the cerebellar proteome to a wild-type-like state. Instead, it produced a disease-context-specific signature dominated by amplification of synaptic, vesicle-trafficking, mitochondrial, and antioxidant pathways beyond both ALS and wild-type baseline levels, accompanied by partial normalization of a smaller RNA splicing and proteasomal stress signature and selective restoration of key synaptic receptors, including the BDNF receptor *Ntrk2*/TrkB. Together, these findings reposition carnosine less as a corrective agent that reverses ALS-associated proteomic pathology and more as a potentiator of endogenous compensatory programs whose magnitude and functional consequences are conditional on the underlying disease state. Establishing whether this hypercompensatory response is protective, neutral, or itself a source of cellular burden will require functional and longitudinal studies beyond the cross-sectional proteomic comparison presented here and will be the focus of future investigations.

## Data Availability

All mass spectrometry proteomics data have been deposited in the Mass Spectrometry Interactive Virtual Environment (MassIVE) under the identifier MSV000101345. During the review process, the data can be accessed using the following credentials: web address: https://massive.ucsd.edu/ProteoSAFe/dataset.jsp?task=325bb8bf017c4f7895a7abd78f222589 (accessed on 31 August 2026); username: MSV000101345_reviewer; password: EIYS9A5R075hsojm.

## Supplementary Materials

The following supporting information can be downloaded at https://www.mdpi.com/article/10.3390/antiox15091117/s1, Figure S1. Carnosine supplementation induces distinct patterns of proteomic remodeling in the cerebellum of ALS rats; Figure S2. Enrichment of mitochondria-related biological processes across experimental comparisons; Figure S3. Gene set enrichment analysis of NRF2-associated pathways and biological processes. Supplementary Information. Modulated proteins by limma comparison.
