# Supplementary material for: Carnosine Potentiates a Compensatory Mitochondrial–Synaptic Proteomic Response in the ALS Cerebellum
Source: Antioxidants (Basel). 2026 Sep 4;15(9):1117. doi: 10.3390/antiox15091117 (PMC13603265; doi:10.3390/antiox15091117)
Supplement: Supplementary file 1 [file antioxidants-15-01117-s001.zip › Supplementary Figures.pdf]

Supplementary Figures

Carnosine Potentiates a Compensatory Mitochondrial–Synaptic Proteomic Response in the ALS Cerebellum

Hellen P. Valerio [1,†], Valeria Oliveira [2,†], Stephanie Y. Ferreira [2], Isabel R. Pereira [2], Giuseppe Palmisano [1], Mariana P. Massafera [2], Vanderson S. Bispo [2], Fernanda M. Prado [2], Paolo Di Mascio [2] and Marisa H. G. Medeiros [2,*]

1 GlycoProteomics Laboratory, Department of Parasitology, ICB, Universidade de São Paulo, São Paulo 05508-000, Brazil; hellen.valerio@usp.br (H.P.V.); palmisano.gp@usp.br (G.P.)
2 Departamento de Bioquímica, Instituto de Química, Universidade de São Paulo, São Paulo 05508-000, Brazil;
valeria.oliveira@usp.br (V.O.); stephanieyaciche@usp.br (S.Y.F.); isabel09@usp.br (I.R.P.); m.massafera-pereira@lesaffre.com (M.P.M.); vanderson.bispo@ephar.com.br (V.S.B.); fmprado@iq.usp.br (F.M.P.); pdmascio@iq.usp.br (P.D.M.)
* Correspondence: mhgdmede@iq.usp.br
† These authors contributed equally to this work.

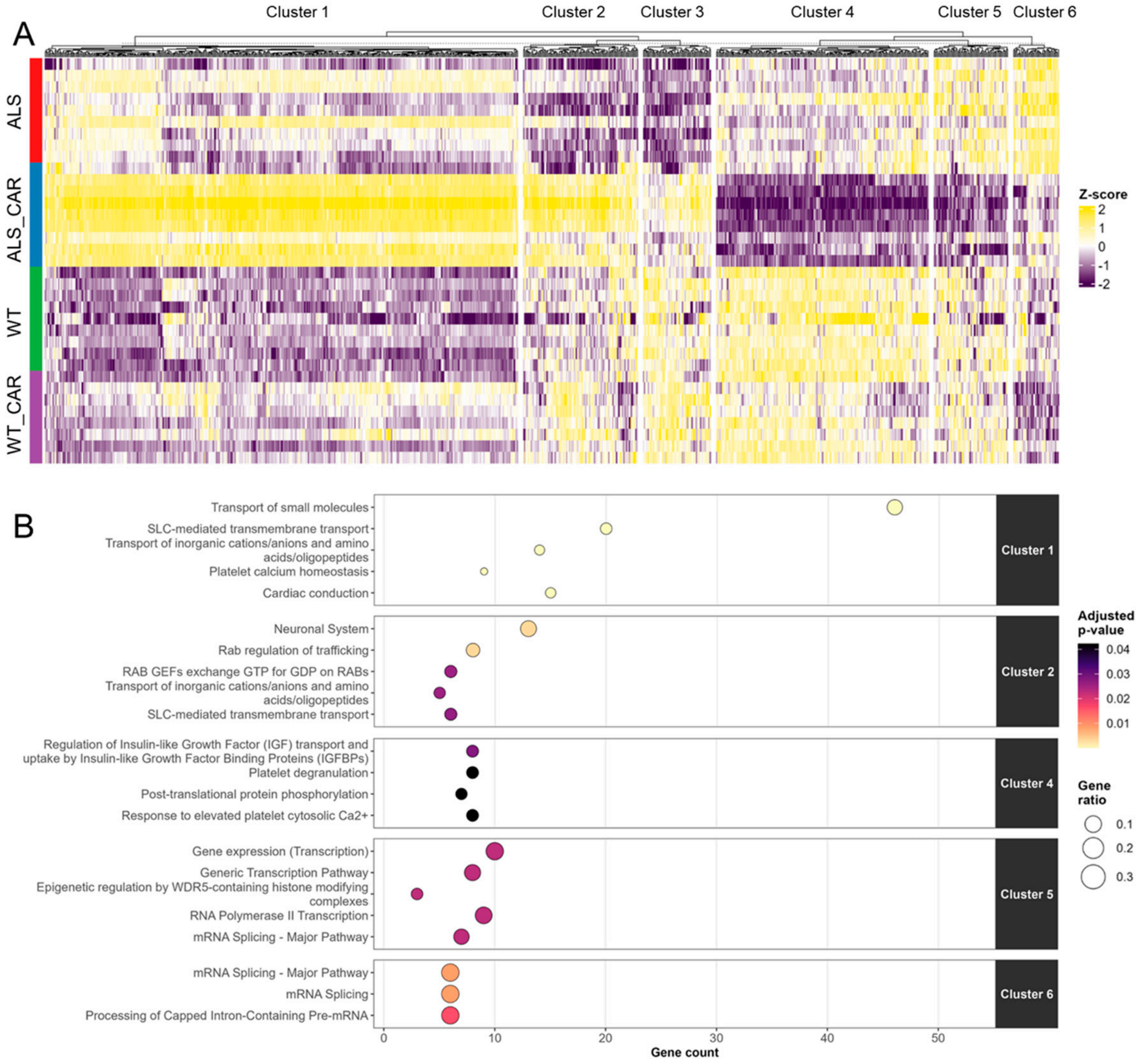

Supplementary Figure S1. Carnosine supplementation induces distinct patterns of proteomic remodeling in the cerebellum of ALS rats. (A) Heatmap showing the relative

abundance profiles of differentially regulated proteins across ALS, carnosine-supplemented ALS (ALS_CAR), wild-type (WT), and carnosine-supplemented wild-type (WT_CAR) animals. Protein abundances were standardized as row-wise Z-scores, with yellow indicating higher and purple indicating lower relative abundance. Proteins were hierarchically clustered and partitioned into six expression clusters, while samples were grouped according to the experimental condition, as indicated by the annotation bar on the left. (B) Reactome pathway enrichment analysis of the proteins assigned to each cluster. The five most significantly enriched pathways with Benjamini–Hochberg-adjusted p-values < 0.05 are shown for each cluster. Dot position represents the number of proteins associated with each pathway, dot size indicates the gene ratio, and dot color represents the adjusted p-value. Only clusters with significant Reactome enrichment are displayed; no significant pathway was identified for Cluster 3.

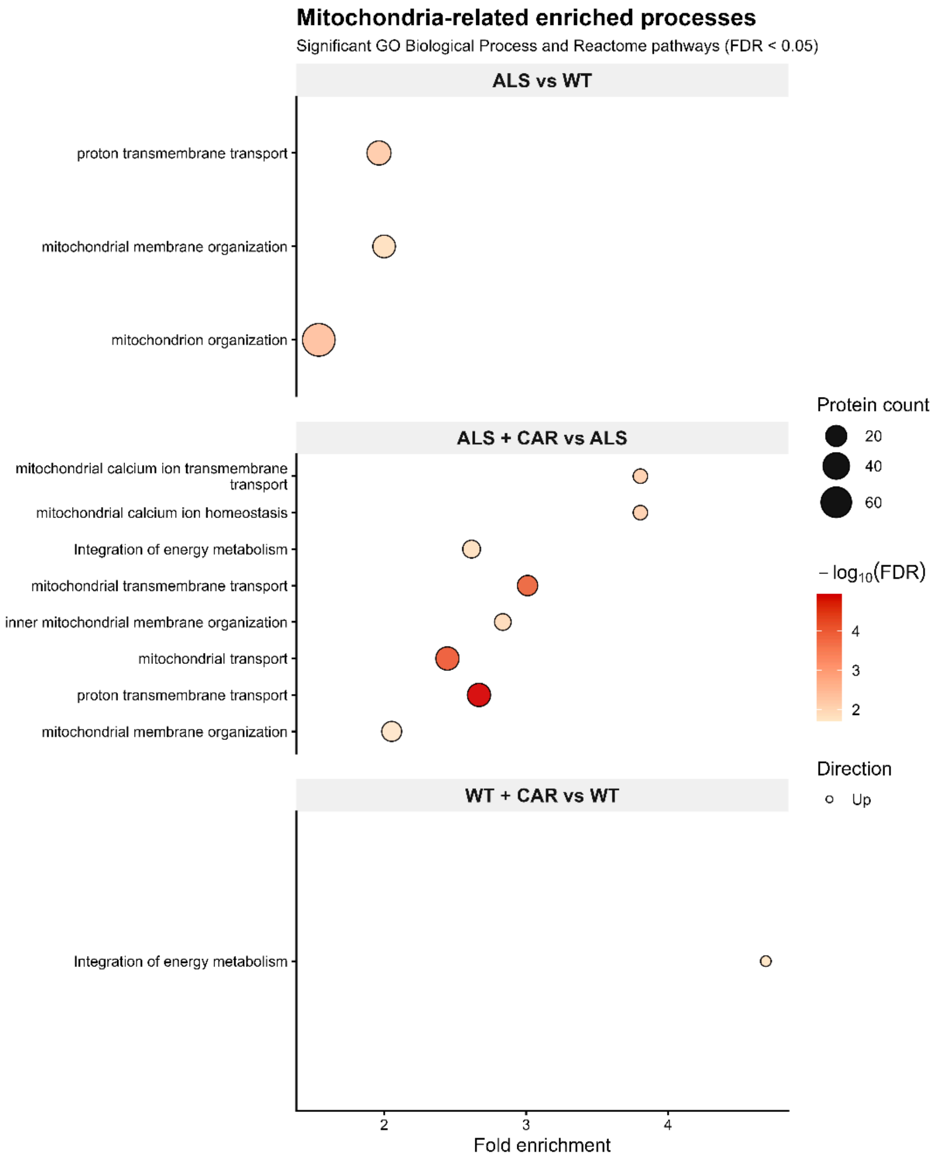

Supplementary Figure S2. Enrichment of mitochondria-related biological processes across experimental comparisons. Dot plots show significantly enriched Gene Ontology Biological Process and Reactome terms associated with mitochondrial function in ALS vs WT,

ALS+CAR vs ALS, and WT+CAR vs WT comparisons. Only pathways with FDR < 0.05 were included. Dot size represents the number of differentially abundant proteins associated with each term, while color indicates enrichment significance as −log10(FDR). The x-axis shows fold enrichment. Symbols indicate whether each process was enriched among upregulated or downregulated proteins in the respective comparison.

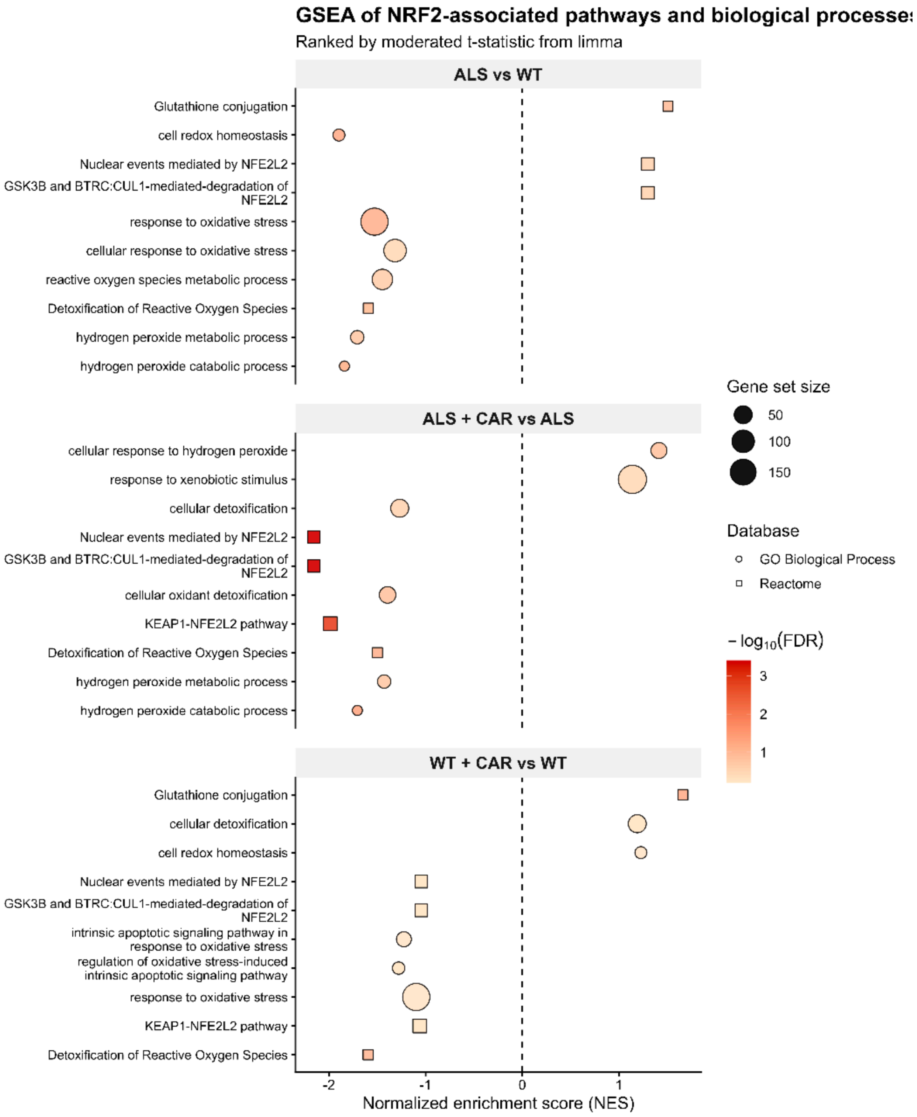

Supplementary Figure S3. Gene set enrichment analysis of NRF2-associated pathways and biological processes. GSEA was performed using the complete proteomic dataset ranked by the moderated t-statistic obtained from limma for the ALS vs WT, ALS+CAR vs ALS, and WT+CAR vs WT comparisons. NRF2-associated terms were selected from Gene Ontology Biological Process and Reactome results, including pathways related to NFE2L2/KEAP1 signaling, oxidative stress, reactive oxygen species detoxification, glutathione metabolism, redox homeostasis, and xenobiotic responses. The x-axis represents the normalized enrichment score (NES), with positive values indicating enrichment toward the first condition of each comparison and negative values indicating enrichment toward the second condition. Point size represents gene set size, color indicates −log10(FDR), and point shape denotes the pathway database. The dashed vertical line indicates NES = 0.
